# Feeling of Guilt, Stress, and Self‐Compassion Among Nursing Staff in the Workplace

**DOI:** 10.1002/nur.70102

**Published:** 2026-07-13

**Authors:** Benedikt Hackert, Anna‐Lena Lumma, Jule Andreas, Ulrich Weger

**Affiliations:** ^1^ Department of Psychology and Psychotherapy Witten/Herdecke University Witten Germany

**Keywords:** emotional burnout, guilt, nursing profession, self‐compassion, stress

## Abstract

Caring for others is a core goal of nursing staff, but providing inadequate care can lead to feelings of guilt which might result in heightened levels of stress. However, self‐compassion might function as a buffer, since it can help to cope with feelings of guilt. A cross‐sectional survey with an exploratory approach was conducted to investigate quantitative associations between guilt and stress, as well as self‐compassion as a potential moderator. Additionally, qualitative data were collected to explore triggers and coping mechanisms related to workplace guilt. A heterogeneous sample of nursing staff (*N* = 179) participated in the survey. Stress, guilt, and self‐compassion were assessed with validated self‐report questionnaires and details about workplace guilt were assessed with open‐ended questions. Interpersonal guilt was linked to increased stress (*r* = 0.41, *p* < 0.01) and less self‐compassion (*r* = −0.59, *p* < 0.01). Moreover, self‐compassion was linked to less stress, too (*r* = −0.65, *p* < 0.01). Against expectations, self‐compassion did not moderate the relationship between guilt and stress. Qualitative findings showed that workplace guilt occurred, on average, 7.26 days per month and was mainly due to shortages of time (37.5%) and staff (18.5%). Coping strategies included communication (30%) and suppression of feelings (9%). Solutions were mainly targeted at system changes (33.5%) and offers for dialog (21.5%). Overall, this study illuminates the underlying dynamics of guilt‐experiences in the workplace and how these experiences are linked to stress and self‐compassion.

## 1 Introduction

The workplace environment of nurses and related healthcare professionals is known to be highly stressful (Babapour et al. 2022). Related to this, feelings of guilt also play a significant role in healthcare (Singhal and Chukkali 2023). Guilt is a moral emotion that can be elicited by moral violations and harm done to others (Tangney et al. 2007). As a result, feelings of guilt can make a person aware of the failures of a particular behavior they have committed and motivate them to make amends or repair the damage done. In some circumstances, guilt can even enhance prosocial behavior (Scaffidi Abbate et al. 2022) and compassion (Lim and DeSteno 2023). However, guilt can also be associated with negative psychological states such as stress, as shown in a previous study of nurses (Dolatshad et al. 2020). In this exploratory study we aimed at better understanding the exact role of feelings of guilt in relation to stress and possible resources for nursing staff to cope with feelings of guilt in the workplace.

Job requirements in healthcare carry a high interpersonal responsibility and range from providing quality care, ensuring patient safety, and administrative work to interpersonal skills. In addition, several occupational stress factors contribute to the highly stressful work environment, including time pressure, an intense workload, and the emotional care of patients. Unfortunately, the cost of this high level of stress often manifests in burnout and other psychological and physical problems (Dall'Ora et al. 2020; Jun et al. 2021; Norful et al. 2024). Increased rates of burnout among nurses and healthcare assistants can, in turn, lead to reduced job performance, including reduced quality of care, absenteeism, or even job‐turnover (Dall'Ora et al. 2015; Dyrbye et al. 2019; Jun et al. 2021; Kelly et al. 2021). Reduced work performance can also result in reduced patient safety and preventable medical errors, which in turn can lead to high overall costs in the healthcare system (Panagioti et al. 2019).

Medical errors, in particular, can be seen as serious stress factors for all types of healthcare workers. A review by Cabilan and Kynoch (2017) showed that the experience of unintentional medical errors also leads to considerable emotional distress in nurses, which is accompanied by feelings of guilt, among other things. Because of these heavy emotional burdens, healthcare workers affected by an unintentional medical error may even be considered second victims (Ozeke et al. 2019; Wu 2000). According to the phenomenon of the second victim, it is not only the patient and their relatives who suffer after a medical error, but also the healthcare worker who involuntarily caused the error (Kappes et al. 2021; Wu 2000). According to a review by Seys et al. (2013), common psychological symptoms that accompany the second victim phenomenon include feelings of guilt. Likewise, Mira et al. (2015) found that among healthcare professionals, including nurses and doctors, feelings of guilt were among the most frequently reported in relation to adverse events.

Other challenges that can arise in the work of healthcare professionals include secondary traumatic stress due to the involvement with patients, some of whom are severely injured and experiencing suffering (Beck 2011). In addition, working with patients requires a high level of emotional and social skills such as emotional care for the patients; as a result, compassion fatigue can occur (Figley 1995; Joinson 1992). Prior research by Duarte and Pinto‐Gouveia (2017) showed that empathy can be linked to increased guilt and thereby increase the risk of burnout and compassion fatigue in nurses. A similar study by Mottaghi et al. (2019) showed that pathogenic feelings of guilt and secondary traumatic stress mediate between empathy and compassion fatigue. Although empathy and emotional care are important in working with patients, these studies show that healthcare workers' socio‐emotional resources can be depleted and that feelings of guilt play an important role in negative states such as stress, burnout, or even compassion fatigue and the second victim phenomenon.

A study by Xue et al. (2022) showed that high psychological capital, including resources such as resilience and hope, can reduce perceived stress and symptoms of PTSD in nurses. Another psychological resource that might benefit healthcare workers is self‐compassion. According to Neff (2023), self‐compassion is accompanied by kindness to oneself, understanding of common humanity, and mindfulness, and can therefore be a more loving way of dealing with challenging situations such as mistakes and potential feelings of guilt. This does not imply that mistakes should be taken lightly; rather, a more constructive and compassionate approach should be adopted for those involved. It has also been shown that self‐compassion can be particularly supportive to cope with feelings of guilt (Valdez and Lilly 2019). Furthermore, several studies have shown that self‐compassion can be beneficial for healthcare practitioners. In a cross‐sectional study with nurses Duarte et al. (2016) showed that self‐compassion can have a protective effect against compassion fatigue. In another cross‐sectional study with nursing students it was shown that self‐compassion could lower perceived stress and thereby reduce levels of anxiety and depression (Luo et al. 2019). In addition, studies have also shown that self‐compassion may have a protective effect against burnout in the healthcare workforce (Abdollahi et al. 2021; Hashem and Zeinoun 2020). Self‐compassion can also be trained through mindfulness‐based and compassion‐based interventions for healthcare professionals, helping to reduce negative work‐related effects (Conversano et al. 2020). Taken together, self‐compassion is an important factor in dealing with feelings of guilt, thereby potentially reducing its negative impact on stress among healthcare professionals.

In this study we aimed at exploring the subjective experience of workplace guilt in nursing staff by combining quantitative and qualitative data. To build on previous research, quantitative data were gathered to investigate the relationship between feelings of guilt, self‐compassion, and stress in healthcare staff. The following hypotheses are theoretically grounded, albeit not formally powered. Thus, we denote them explicitly as working hypotheses and follow an exploratory approach. On the basis of the studies mentioned above, according to which feelings of guilt are associated with increased risk of burnout and compassion fatigue in nurses, we expect that feelings of guilt are related to an increase in perceived stress (working hypothesis a). Since studies show that self‐compassion is associated with less stress in nurses, we also expect that more self‐compassion is associated with less perceived stress (working hypothesis b). Moreover, based on studies showing that self‐compassion can have a protective effect against several negative emotions, we expect that greater self‐compassion is related to decreased feelings of guilt (working hypothesis c). Finally, we expect that the effect of feelings of guilt on the experience of stress is moderated by self‐compassion (working hypothesis d). To explore the role of guilt in nursing stuff more deeply, open‐ended questions were used to explore situations in which workplace guilt arises and how participants cope with it. In addition, suggestions for improved coping with such guilt were assessed.

## 2 Methods

### 2.1 Study Design

Data were collected with an online survey employing a correlational research design with a multi‐methods approach. Quantitative data were used to investigate relationships between (work) guilt, self‐compassion, and stress. Qualitative data were used to explore triggers of as well as coping with workplace guilt.

### 2.2 Participants

This cross‐sectional survey was carried out with a German‐speaking sample. We did not assess cultural background as this was not the primary focus of our study. Participants were recruited via social media, notices at Witten/Herdecke University (Germany), and medical clinics. A heterogeneous sample of healthcare staff working in inpatient care was analyzed (see Table 1). Participation in the study was voluntary and did not include financial compensation. The study was conducted in Germany from December 28th, 2022, till February 2nd, 2023.

**Table 1:** Overview of educational level, types of professional education, and types of workplaces.

Category	Frequency	Percentage
Type of professional education^a^		
Registered nurse^b^	151	84.36%
Higher (e.g., advanced studies, director of nursing)	11	6.15%
Certified nursing assistant c	10	5.59%
None/other	7	3.91%
Type of workplace		
Hospital	82	45.81
Nursing home	74	41.34
Other	12	6.71
Psychiatry	11	6.15
Educational level^d^		
Intermediate or secondary school certificate^e^	107	59.78
Vocational diploma^f^	45	25.14
Advanced college entrance qualification^g^	19	10.61
Bachelor's or Master's degree	8	4.47

^a^ The majority of our sample got their training in Germany according to a now outdated educational system for nurses which has been restructured substantially in 2020. Keeping differences in educational systems between countries in mind, we use international labels for comparability (e.g., Fassmer et al. 2025; Praxmarer‐Fernande et al. 2017). Registered nurses include the German job roles “Gesundheits‐ und Krankenpfleger” and “Altenpfleger.”

^b^ The U.S. equivalent is professional nurse.

^c^ The U.S. equivalent is assistive personnel.

^d^ Typical equivalencies for U.S./England are as following:

^e^ GCSEs/High School Diploma.

^f^ AS‐Levels/High School Diploma.

^g^ A‐Levels/High School Diploma + AP courses.

A total of 290 participants enrolled in the survey, but several participants did not complete the full survey. Only participants who were at least 18 years old, working in an inpatient care setting (e.g., in a hospital, nursing home, or psychiatric facility), and who had at least 1 year of work experience, were included in the analyses. In total, nine participants were excluded from the final analysis; eight participants did not indicate that they work in an inpatient setting and one participant did not indicate how long they worked in the healthcare setting. For the main hypotheses only data were used of those participants, who at least completed all three questionnaires. A total of 229 participants filled out the Perceived Stress Scale, a total of 196 participants filled out the Self‐Compassion Scale and a total of 188 people filled out the Interpersonal Guilt Questionnaire. A total of 188 participants provided complete data for all three questionnaires and were screened for inclusion criteria. The final sample that met all inclusion criteria, consisted of 179 participants.

### 2.3 Procedure and Data Collection

Limesurvey was used as a platform to carry out the online survey (LimeSurvey n.d.). Prior to participation, participants gave informed consent. Participants were then asked to provide demographic information, including age, gender, level of education, and information about their professional training. Next, participants were asked to complete the Interpersonal Guilt Questionnaire, the Self‐Compassion Scale, and the Perceived Stress Scale. Finally, participants were asked to provide written answers to four open‐ended questions. They were also able to provide voluntary comments or feedback on the study.

### 2.4 Measurement Section

#### 2.4.1 Interpersonal Guilt

Subjective experience of interpersonal guilt was assessed with the Interpersonal Guilt Questionnaire (IGQ) originally developed by O'Connor et al. (1997) to “assess guilt related to concern about harming others” (Abstract). For this study the German short form of the IGQ (Albani et al. 2002) was used, which has 21 items and three subscales. The Separation‐/Disloyalty subscale pertains to a feeling of guilt resulting from separation from significant others and pursuing own goals, and assuming this might hurt them (Albani et al. 2002; O'Connor et al. 1997). An example item is: “It makes me uncomfortable to do things differently than my parents did.” Survivor guilt contains the belief that one's success is unfair to those who have not attained the same and that it makes them feel bad, reflecting an irrational sense of inequity (O'Connor et al. 1997). An example item is: “I feel uncomfortable when I am more successful than my friends or family.” Omnipotent Responsibility Guilt includes items that describe excessive concern for (the well‐being of) others (Albani et al. 2002). An example item is: “I care a lot about the welfare of people I like, even if they are well.” The scale has been referenced in related studies in the health‐care context (e.g., Duarte and Pinto‐Gouveia 2017; Mottaghi et al. 2019).

Response options range on a 5‐point Likert scale from 1 (*strongly disagree*) to 5 (*strongly agree*). The internal consistency of the full scale was reported for a sample consisting of alcohol dependent in‐patients and healthy controls (Cronbach's alpha α = 0.88; Braun et al. 2018). For the statistical analyses, a mean score on the total scale was computed (range from 1 to 5, see above). A higher IGQ score means the participant perceived greater interpersonal guilt.

#### 2.4.2 Self‐Compassion

Self‐compassion is “a positive stance toward oneself when things go badly“ (Hupfeld and Ruffieux 2011, Abstract). The Self‐Compassion Scale (SCS) assesses such stance along six subscales (Neff 2003). The German short version of the Self‐Compassion Scale, used in this study, includes 12 items and all six subscales (Hupfeld and Ruffieux 2011; Raes et al. 2011). Response options range from 1 *(almost never*) to 5 (*almost always*) on a 5‐point Likert scale. Sample items for each subscale are “When I'm going through a very hard time, I give myself the caring and tenderness I need.” (Self‐Kindness); “I'm disapproving and judgmental about my own flaws and inadequacies” (Self‐Judgment); “When I feel inadequate in some way, I try to remind myself that feelings of inadequacy are shared by most people.” (Common Humanity); “When I'm feeling down, I tend to feel like most other people are probably happier than I am.” (Isolation); “When something upsets me I try to keep my emotions in balance.” (Mindfulness); “When I'm feeling down I tend to obsess and fixate on everything that's wrong.” (Over‐Raes et al. 2011; Appendix). The internal consistency of the complete scale pertaining to a German‐speaking sample with interest in psychological online research was excellent (α = 0.91; Hupfeld and Ruffieux 2011). For the statistical analyses, items pertaining to the subscales Self‐Judgment, Isolation, and Over‐Identification were reverse‐coded and a mean score on the total scale was computed (range from 1 to 5, see above; Hupfeld and Ruffieux 2011). A higher SCS score indicates a higher level of self‐compassion.

#### 2.4.3 Stress

The Perceived Stress Scale (PSS) by Cohen et al. (1983) was used to assess “the degree to which situations in one's life are appraised as stressful” (p. 387). For this study, the German translation was used (Klein et al. 2016). Response options range from 1 *(never*) to 5 (*very often*) on a 5‐point Likert scale. The PSS has two subscales including the Perceived Helplessness subscale with 6 items (e.g., “In the last month, how often have you felt nervous and stressed?”) and the Perceived Self‐Efficacy subscale with four items (e.g., “In the last month, how often have you felt confident about your ability to handle your personal problems?”). The full scale demonstrated good internal consistency when applied to a representative German community sample (α = 0.84; Klein et al. 2016). For the statistical analyses, positively‐valenced items were reverse‐coded and a total score was computed by summing up all items (range from 10 to 50). A higher PSS score indicates a higher level of perceived stress (i.e., worse outcome; Klein et al. 2016).

#### 2.4.4 Workplace Guilt

After completing the questionnaires, participants were asked “*How often do you experience guilt due to situations in your work life?*” and to indicate the number of days per month. Following, participants answered three open‐ended questions to gain an in‐depth understanding of how health care professionals deal with the experience of guilt in the workplace: “Please describe a typical situation of your work life that induces experiences of guilt in you. “How do you cope with feelings of guilt?”; “What do you think is needed to better cope with feelings of guilt in your work context?”

### 2.5 Data Analysis

Prior to testing the statistical hypotheses, all main variables were screened for outliers. Before conducting moderation analyses, heteroskedasticity was tested with the Breusch Pagan Test and linearity was checked with visual inspection of scatterplots after LOESS smoothing. Internal consistency for the whole scales was assessed using Cronbach's alpha. Since all analysis procedures were robust against violations of normality, we did not include information on it. Pearson correlations were used to assess the linear relationships between approximately continuous variables (i.e., IGQ, SCS, PSS, age, and work experience) whereas a Spearman correlation was used for ordinal variables (i.e., level of education). In addition, a Welch ANOVA was conducted to test for differences in stress levels between fields of work.

To test for the moderating effect of self‐compassion between stress and interpersonal guilt, the PROCESS macro was used to run a moderation analysis (Hayes 2022) with guilt as the independent variable, stress as the dependent variable and self‐compassion as the moderator―similar to other studies that have used self‐compassion as a moderating variable (cf. Mróz and Sornat 2023; Samaie and Farahani 2011). All statistical analyses were performed using SPSS (Version 29, IBM‐Corp 2023).

One of the authors inductively formed categories from the open‐ended responses in order to reduce and structure the data material following a systematic screening similar to a content analysis (Rädiker and Kuckartz 2019). In the following we list the categories for each of the three open‐ended questions (see Supporting Information S1 for descriptions and examples for each code):

- Guilt‐triggering situations: personnel shortage, time shortage, unaccomplished tasks, errors, lack of assistance, unexpected situations, dying process, taking time for yourself, no feelings of guilt, other, no valid entry
- Strategies for coping with guilt at work: reflection, apologies, communication, self‐compassion/acceptance, separate private & professional life, distraction/compensation, suppress, negative emotions, think/ruminate, harmful behavior, no feelings of guilt, other, no valid entry
- New ways to deal with guilt in the workplace: system changes, offers for dialog, support, appreciation & understanding, further education, opportunity for reflection, find balance, acceptance of the situation and mistakes/self‐compassion, no feelings of guilt/no suggestions, other, no valid entry

Based on these inductively formed categories, rating manuals were created for two independent raters, including sample statements and how they would be related to a respective category (Rädiker and Kuckartz 2019). A description could fall into more than one category. The two independent raters checked the data material and categorized the open answers accordingly. We averaged the number of codings by the two raters to indicate the frequency of its occurrence. Inter‐rater‐agreement was assessed using Kohen's Kappa κ (Halpin 2024; see Supporting Information S3 for information on inter‐rater‐agreement). The threshold for codes to be included in the results section was set to κ ≥ 0.8 (McHugh 2012).

### 2.6 Ethical Considerations

The study was approved by the Ethics Committee of Witten/Herdecke University (S‐256/2022) and was conducted in accordance with the Declaration of Helsinki. All participants were informed about the purpose of the study and gave informed consent before completing the questionnaires. Participants could withdraw from the study at any time without giving a reason.

## 3 Results

The mean age of the final sample was *M* = 37.5 (standard deviation [SD] = 10.4) with a minimum age of 21 and a maximum age of 62. The majority of participants was female (*N* = 171), seven participants were male, and one participant did not indicate their gender. Demographic information on the highest educational qualification of the participants and type of workplace is given in Table 1. The average work experience in years was *M* = 15.44 (SD = 9.57) with a minimum of 1 and a maximum of 43 years of work experience. Descriptive results for the total sample showed a mean of *M* = 3.03 for self‐compassion (SD = 0.72), *M* = 2.83 for interpersonal guilt (SD = 0.75), and *M* = 30.8 for stress (SD = 6.24).

### 3.1 Correlations of Guilt, Self‐Compassion, and Stress

Cronbach's alpha for the whole scales indicated good reliability (all αs = 0.88).

Results from the Pearson correlation analyses (see Table 2) indicated a moderate positive correlation between feelings of guilt and stress (*r* = 0.41, *p* < 0.01), confirming working hypothesis a. Self‐compassion was moderately negatively correlated to feelings of guilt (*r* = *‐*.59, *p* < 0.01) and stress (*r* = *‐*.65, *p* < 0.01), confirming working hypotheses b and c.

**Table 2:** Correlation coefficients between interpersonal guilt, self‐compassion, perceived stress, and guilt experienced at work.

Variable	1	2	3	4
1. IGQ	—	—	—	—
2. SCS	−0.59*	—	—	—
3. PSS	0.41*	−0.65*	—	—
4. work guilt	0.35*	−0.32*	0.38*	—

*Note:* IGQ, Interpersonal Guilt Questionnaire; SCS, Self‐Compassion Scale; PSS, Perceived Stress Scale; work guilt = days per month guilt experienced at work.

* *p* < 0.001.

#### 3.1.1 Frequency of Guilt Experience Per Month

Of the 179 participants, 130 provided a reply to the question “How frequently do you experience guilt at work?” Participants' experience of guilt during work in the healthcare setting occurred, on average, *M* = 7.26 (SD = 7.58) days per month with a minimum of 0 days and a maximum of 31 days. Correlational analyses (see Table 2) revealed that the frequency of the monthly experience of guilt was positively correlated with interpersonal guilt (*r* = 0.35, *p* < 0.01) as well as stress (*r* = 0.38, *p* < 0.01) and, furthermore, negatively correlated with self‐compassion (*r* = *‐*.32, *p* < 0.01). These associations can be considered weak to moderate.

#### 3.1.2 Correlations With Demographical Data

In addition, we explored relations between demographical variables and stress. Pearson correlation analyses showed no significant correlations between stress and work experience (in months, *p* = 0.20) or age (*p* = 0.09). Only a higher education was slightly correlated with lower stress (*r*_*s*_ = ‐0.17; *p* = 0.03) as indicated by a spearman rank order correlation analysis. Furthermore, a Welch ANOVA found no effect of field of work on stress (*p* = 0.57).

### 3.2 Moderation

**Figure 1:**
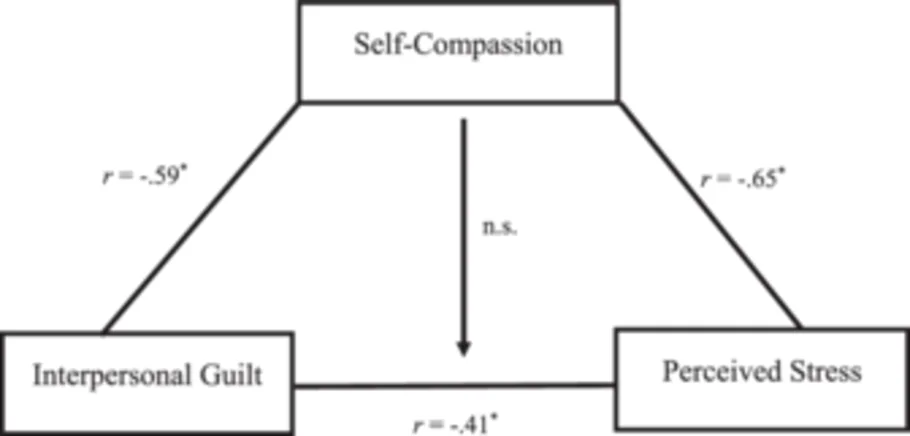
Correlational model. *Note:* *p < 0.001.

Visual inspection of the scatterplots after LOESS smoothing indicated that all variables used in the moderation analysis were approximately linear (see Supporting Information S1: Figure 1). Heteroskedasticity was tested with the Breusch Pagan Test (*p* = 0.12).

A moderation analysis using robust HC3 standard errors was run to check whether self‐compassion moderated the effect of the experience of guilt on stress (statistically speaking, that the regression coefficient of the interaction term FIS×SCS ≠ 0). The overall model was significant, Fisher's F ratio *F*(3,175) = 36.67, *p* < 0.01, predicting 43.29% of the variance. The moderation analysis showed that self‐compassion did not moderate the effect between guilt and stress (increase in explained variance 0.39%, *F*(1, 175) = 0.44, *p* = 0.51), disconfirming working hypothesis d. Further results of the moderation analysis are shown in Figure 1 and Table 3.

**Table 3:** Results for moderation analysis.

Variable	*B*	*SE (HC3)*	*t*	*p*	*CI*
IGQ	−0.54	1.01	−0.53	0. 60	−2.54, 1.46
SCS	−7.19	2.51	−2.86	< 0.01	−12.15, −2.22
IGQ×SCS	0.21	0.32	0.66	0.51	−0.42, 0.84

Abbreviations: IGQ, Interpersonal Guilt Questionnaire; SCS, Self‐Compassion Scale; B, regression coefficient; SE, standard error; CI, confidence interval.

### 3.3 Qualitative Analysis of Workplace Guilt

The most frequent guilt trigger was time shortage mentioned with a frequency of 37.5%. It included delivering only the most essential care and too little time to listen to patients' concerns. Closely intertwined with this was personnel shortage (18.5%) resulting in feelings of guilt because, for instance, nurses must weigh up which patient should be given priority. To a lesser degree, medical errors (9.5%) like failing to recognize an emergency or dealing with the dying process (8%) were mentioned. About a third of all coping behaviors were communicative acts (30%), especially talking about the incident with colleagues, partners, or family members. Related to this, apologizing or explaining directly to the patient was named with a frequency of 5%. Emotion‐focused coping entailed detrimental behaviors like suppression of feelings (9%) and rumination (7.5%) but also self‐compassion and acceptance (8.%), for example through positive self‐talk. Suggestions for improvement most often aimed at structural changes in the health care system (33.5%), ranging from higher salaries and staff ratios to work tasks better aligned with one's area of competence, as well as ideological aspects of profit and appreciation of nurses. Moreover, offers for (interdisciplinary) dialog (21.5%) and opportunities to reflect on straining situations (6.5%) were wished for.

The frequency of occurrence of distinct guilt‐triggering situations, strategies to cope with workplace guilt as well as suggestions for improved ways to deal with workplace guilt in the answers to the open‐ended questions in the total sample with at least good inter‐rater‐agreement (i.e., κ ≥ 0.8; McHugh 2012) are shown in Table 4. In terms of content, descriptions of the codes along with exemplary quotes can be found likewise in Table 4.

**Table 4:** Qualitative findings pertaining to workplace guilt.

Maincode	Frequency	Example quotation	Scope of topics based on individual answers of participants
Subcode	(κ)		
Guilt‐Triggers			
Personnel shortage	18.5% (*κ* = 0.92)	*“A typical everyday situation is that you have to cut corners with virtually every care task because there isn't enough time.” (ID 165)*.	Too many patients per nurse; overburdening; inadequate care (physical and interpersonal); weighing up which patient should be given priority; unable to complete all tasks
Time shortage	37.5% (*κ* = 0.86)	*“Despite our best efforts, we have to leave people lying in their own excrements… Patients die alone without our assistance… The only thing that matters is profit.” (ID 259)*	No time due to high workload or staff shortages; only the most essential care; work must be done quickly; too little time for patients/relatives/to listen to concerns and needs/guidance of patients or trainees/tasks; long waiting times for residents; skip breaks; exhaustion
Errors	9.5% (*κ* = 0.82)	*“Only if I have actually messed something up.” (ID 243)*	fail to recognize an emergency or symptom; misjudging a situation; forget something; mistake made at the expense of the patient due to systemic shortage of nursing staff
Dying process	4% (*κ* = 1.0)	*” When I have to reanimate patients even though they didn't really want it.” (ID 188)*	Unsuccessful resuscitation of a dear patient; no time to accompany the dying process
No feelings of guilt	8% (*κ* = 0.92)	*“I never have feelings of guilt at work.” (ID 168)*	no (more) feelings of guilt
Coping Strategies			
Apologies	5% (*κ* = 0.85)	*“I apologize to the patients for the situation and explain it to them.” (ID 146)*	Apologize (repeatedly) to the patient; explain (e.g., situation, waiting times); justify oneself; talk openly about it; make up for it
Communication	30% (*κ* = 0.96)	*“I talk to work colleagues or my husband about the situation.” (ID 195)*	Talk about the incident (e.g., with coworkers, family, partner, friends); open communication; keep it to oneself; convince management/colleagues
Self‐compassion/acceptance	8% (*κ* = 0.9)	*“I try to tell myself that I do what I can.” (ID 217)*	Self‐talk (e.g., I am doing what I can, everyone can forget something, I can't change the situation); accept; feelings of guilt come and go
Distraction/compensation	8% (*κ* = 0.85)	*“Distract myself.” (ID 272)*	Distraction; find balance (e.g., with music or sports); go home and switch off; clear head; read; play videogames; clean; occupy oneself with other things
Suppress	9% (*κ* = 0.96)	*“Put up with it, push it away after work and ignore it. I don't know any other option.” (ID 165)*	suppress feelings; endure/push away/ignore feelings of guilt; slowly block them out; need to be alone
Think/ruminate	7.5% (*κ* = 0.83)	*“Think a lot, even at home.” (ID 108)*	Think about it often (also after work); take everything home; mind can't switch off; sadness and anger; wonder if things will get better; brood; dreams about work; consumption of too much alcohol
Improvements			
System changes	33.5% (*κ* = 0.88)	*“I am firmly convinced that these 'feelings of guilt' only arise due to the given framework conditions. It would simply take fundamentally different structures to prevent them from arising in the first place.” (ID 245)*	More (dedicated) staff (e.g., service staff who distributes food, staff who does the cleaning, secretaries who take care of all the admissions, discharges and registrations); higher staffing ratios; more time (e.g., for patients); more supervision; better healthcare system; fundamentally different structures; good shift planning; no constant shift changes (preferably block shifts); better pay; less pressure to save money due to the greed of the top bosses; more discussions need to be held with employees; more appreciation of the profession; no work beyond one's area of competence; more security
Offers for dialog	21.5% (*κ* = 0.91)	*“Offers of conversations from uninvolved persons.” (ID 276)*.	relieving conversations; mutual exchange; someone available to talk to; trusted people who listen to you; continued talking to colleagues; open communication; opportunities to talk about feelings of guilt; feeling of being able to speak openly; team discussions; supervision; psychological support for staff from the employer; coaching; collegial consultation; opportunity to exchange ideas with colleagues (not during handover); more team meetings; open communication between professional groups; team‐building measures
Opportunity for reflection	6.5% (*κ* = 0.81)	*“Reflect on serious situations with superiors and colleagues.” (ID 211)*	self‐reflection (e.g., reflect on working day and approach it differently the next day); discussion and reflection with the team (e.g., on serious situations); (team‐)supervision (e.g., following traumatic events like a frustrating resuscitation of a newborn; psychological support for staff by the employer; training in systemic counseling to learn methods to deal with yourself better; extra time for reflection; receiving praise from supervisors
Find balance	2% (*κ* = 0.8)	*“Work‐life balance.” (ID 73)*	Work‐life balance; ease

*Note*: Frequencies of codings were averaged across the two raters. Threshold for inter‐rater agreement was set to *κ* = 0.8.

## 4 Discussion

The aim of this study was to investigate eliciting situations and coping strategies of workplace guilt among nursing staff and the relationship between workplace guilt, stress, and self‐compassion. Quantitative findings from this study confirmed our hypothesis that greater feelings of guilt are associated with increased stress levels in nursing staff (working hypothesis a). This is consistent with previous studies showing a link between the experience of pathogenic guilt and compassion fatigue or secondary traumatic stress (Duarte and Pinto‐Gouveia 2017; Mottaghi et al. 2019). As expected, more self‐compassion was linked to less feelings of guilt and less perceived stress (working hypothesis b and c). This is in line with previous studies showing that self‐compassion is related to less negative psychological states in nursing professionals such as stress, burnout, or compassion fatigue (Abdollahi et al. 2021; Duarte et al. 2016; Hashem and Zeinoun 2020; Luo et al. 2019). Taken together, these findings corroborate existing research showing the detrimental effects of stress and negative emotions on healthcare workers, underscoring the importance of effective coping strategies (Babapour et al. 2022; Norful et al. 2024). Participants from our sample reported feelings of guilt, on average, 7 days per month, which is in line with the frequency reported by Mira et al. (2015).

The hypothesis that the relationship between guilt and stress is moderated by self‐compassion, however, was not confirmed (working hypothesis d). The moderating effect of self‐compassion on the relationship between guilt and perceived stress was not significant. This may be because other factors are more relevant in the relationship between guilt and stress. For example, guilt might only increase stress under certain conditions, such as reduced emotion regulation or negative rumination patterns. Thus, a model that integrates additional psychological factors such as adaptive versus maladaptive coping with guilt may provide a more comprehensive understanding of the relationship between feelings of guilt and stress. Interestingly, in a study by Dev et al. (2020) it was shown that self‐compassion strengthened the relationship between stress and burnout in nurses. Another study showed that self‐criticism was a better predictor of compassion fatigue than self‐compassion in helping professions including nurses (Ondrejková and Halamová 2022). Self‐criticism can also be accompanied by feelings of guilt, so it could be that this is also a decisive factor in the connection between feelings of guilt and stress. Future studies could investigate in a long‐term study whether training can increase self‐compassion and investigate whether training‐induced self‐compassion moderates the relationship between feelings of guilt and the experience of stress (Ruiz‐Fernández et al. 2020; Wasson et al. 2020).

The qualitative data provided in‐depth and bottom‐up information from the perspective of the nurses. Situations that led to feelings of guilt in the workplace mainly related to a lack of resources such as time shortage and lack of staff. Such responses indicate that primarily structural and systemic conditions trigger feelings of guilt, highlighting calls from previous studies that working conditions in the healthcare sector urgently need to change (Norful et al. 2024). For example, the restrictive structures of the health care system may make it difficult for nurses to apologize to patients after mistakes or to provide adequate care, thus damaging interpersonal relationships with patients and preventing feelings of guilt from improving prosocial behavior and compassion (Scaffidi Abbate et al. 2022). These findings indicate that the solution for dealing with guilt cannot lie with the individual alone (e.g., by participating in psychological interventions), but that structural changes in the health care system are also needed (Valdez 2024). Even minor changes might be helpful. For instance, short breaks and a room for interpersonal reflection about adverse events during a working day might be helpful, since talking with colleagues or acquaintances was by far the most common strategy to alleviate feelings of guilt. Still, participants' voiced need for support, appreciation, and understanding must also be addressed on an individual‐psychological level; for instance, through psychological interventions (Conversano et al. 2020) targeted at better management of feelings of guilt and through trauma‐informed care (Lewis et al. 2019; Selwyn and Lathan 2021). As one starting point for such interventions, reports from our sample about repressed feelings could be used.

### 4.1 Limitations and Recommendations for Future Research

There are some limitations to this study, but also possible improvements for future research on this topic. Firstly, the heterogeneity of the sample in terms of type of workplace, work experience, or educational level, may have masked associations in subpopulations. For instance, a psychiatry ward may pose different (systemic) challenges than an elderly home and, thus, the practicability and usefulness of self‐compassion may differ. To this regard, a mixed‐methods approach (i.e., an integration of qualitative with quantitative data instead of only parallel use of both) may be helpful, since some forms of self‐compassion might be more suited for certain causes of guilt. For example, personal mistakes might be countered with self‐compassion while guilt resulting from unfinished tasks caused by a shortage of staff might not. This might explain, why workplace guilt had only a weak correlation with SCS. In addition, the sample consisted mainly of female participants, which could also have had an influence on the questionnaire responses. Furthermore, this survey was conducted in Germany and the cultural background of the study sample was not assessed. Follow‐up studies should also consider the cultural context, including religious differences. Pre‐existing mental illness may also influence guilt and stress in healthcare workers and should be considered as an additional variable or exclusion criterion in future studies.

Temporal progression could be another factor that plays an important role in analyzing the relationship between guilt and stress, as guilt may precede stress, or stress may precede guilt, or they may influence each other in a bidirectional way. For example, it has been shown that work overload and psychological exhaustion precedes feelings of guilt (Figueiredo‐Ferraz et al. 2021). In the context of PTSD, experimentally induced self‐blame for a stressor led to more guilt feelings which were in turn followed by heightened stressful intrusive thoughts (Bub and Lommen 2017). Relating this to our findings, nurses felt guilty for being inattentive to patients' symptoms (due to time pressure) which in some cases caused problems.

In addition, the questionnaire used in this study might not be specific enough to capture feelings of guilt that are representative of the feelings of guilt experienced at work; this might explain the rather weak correlation between IGQ and work guilt. It may be useful to use questionnaires representing constructs that are specifically tailored to the daily work context of nursing staff (e.g., the Caregiver Guilt Questionnaire; Losada et al. 2010). However, IGQ has certainly been shown to be relevant for the nursing context, albeit previous research has focused on the subscales Survivor Guilt and Omnipotent Responsibility Guilt (Duarte and Pinto‐Gouveia 2017; Mottaghi et al. 2019). The Separation/‐Disloyalty subscale, on the other hand, focuses on the parent‐child relationship in most of its items and, thus, might insufficiently represent the relationship between caregivers and patients (e.g., private vs. professional context; parents vs. caregivers as authority figures). Furthermore, survivor guilt might be especially relevant in contexts where death occurs frequently (e.g., oncology, palliative care, or intensive care unit nurses). As to the authors' knowledge, no data exists to date on differences in survivors' guilt between different nursing roles.

Correlations between frequency of work guilt and PSS were weak to moderate. Similar to the guilt measure, the stress measure could have been specified to the working context of nurses. Moreover, stress might be largely dependent on the intensity of the guilt feeling and only partly determined by its frequency. In addition, whether a guilt‐feeling was only short‐lived and coped with immediately, or whether it lasts for several days could be of further relevance. To this regard, intensity of work guilt, duration of guilt, and rumination about the guilt‐inducing event might be worth to explore.

Statistically, workplace guilt was measured with a single item scale which can be problematic in terms of reliability and validity (Allen et al. 2022). Furthermore, no definition of guilt was provided for the participants. Another source of measurement error concerning workplace guilt might be that participants rather counted days of guilt‐inducing events than any days on which guilt was experienced. Taken together, such measurement error might attenuate the strength of the correlation with any of the major variables (e.g., Goodwin and Leech 2006; Muchinsky 1996).

Related to the above is the need for a more precise definition of the concepts to be analyzed, thus creating a good conceptual clarity, which is sometimes lacking in psychology (Scheel 2022). To this regard, qualitative descriptions from our data could be used to adopt specific subscales from, for example, the Caregiver Guilt Questionnaire (e.g., Guilt About Doing Wrong by the Care Recipient, Guilt About Failing to Meet the Challenges of Caregiving, Guilt About Neglecting Others; Losada et al. 2010). Likewise, more precise and time‐sensitive methods, such as experience sampling, may be useful for further studies. For example, an experience sampling study could be used during daily work to capture feelings of guilt in a more ecologically valid way. In addition to the subjective experience of guilt during work, physiological parameters could also be recorded through ambulatory assessment (Trull and Ebner‐Priemer 2014).

For an in‐depth exploration of a concrete experience of guilt in the work context, micro‐phenomenology would also be a suitable method (Petitmengin 2006). A micro‐phenomenological interview could be used to investigate a specific moment of guilt at work, thereby gaining new insights into the temporal unfolding of the feeling of guilt. In the long term, this information could be used, for example, by healthcare staff to identify the onset of guilt at an early stage, which could help them to let go of guilt more easily. Finally, conducting longitudinal studies could lead to a better understanding of the causal relationships between stress, guilt, and self‐compassion in healthcare professionals.

## 5 Conclusion

Overall, findings from this study show that workplace guilt of nursing staff is common and provide insights into the work‐related aspects that cause guilt and how nursing staff cope with these feelings. Suggestions for better coping with guilt at work relate to structural factors, but also to communication. Our study also provides further evidence that feelings of guilt and the frequency of guilt experienced at work in nurses is related to greater levels of stress. Conversely, self‐compassion was associated with less feelings of guilt and stress in this sample. However, future research is needed to elucidate the factors that promote guilt with adaptive or maladaptive states and the role of self‐compassion in the context of guilt and related stress. In the long term, a better understanding of the links between feelings of guilt and stress in the workplace for nurses may contribute to the improvement of working conditions.

## Author Contributions

All four authors qualify for authorship. Each author has contributed to the various stages to varying degrees, that is, substantial contributions to the conception or design of the work; or the acquisition, analysis, or interpretation of data for the work; or drafting the work or reviewing it critically for important intellectual content. All individuals had the opportunity to participate in the review, drafting, and final approval of the manuscript.

## Funding

The authors have nothing to report.

## Ethics Statement

This study was approved by the Ethics Committee of Witten/Herdecke University (application no. S‐256/2022).

## Consent

Prior to participation, participants were informed of the purpose of the study and were required to give written informed consent before participating in the study.

## Conflicts of Interest

The authors declare no conflicts of interest.

## Supporting information


Supporting File

## Data Availability

The data analyzed in this article will be made available by the authors upon reasonable request and will be provided without undue reservation.

## References

[nur70102-bib-0001] Abdollahi, A. , A. Taheri , and K. A. Allen . 2021. “Perceived Stress, Self‐Compassion and Job Burnout in Nurses: The Moderating Role of Self‐Compassion.” Journal of Research in Nursing 26, no. 3: 182–191. https://www.ncbi.nlm.nih.gov/pmc/articles/PMC8894998/pdf/10.1177_1744987120970612.pdf. 35251240 10.1177/1744987120970612 PMC8894998

[nur70102-bib-0002] Albani, C. , G. Blaser , A. Körner , et al. 2002. “Der Fragebogen zu interpersonellen Schuldgefühlen” (FIS) [The German Short Version of the Interpersonal Guilt Questionnaire].” Anwendung in einer repräsentativen Bevölkerungsstichprobe und bei PsychotherapiepatientInnen 52, no. 03/04: 189–197. 10.1055/s-2002-24952. 11941527

[nur70102-bib-0003] Allen, M. S. , D. Iliescu , and S. Greiff . 2022. “Single Item Measures in Psychological Science: A Call to Action.” European Journal of Psychological Assessment 38: 1–5. 10.1027/1015-5759/a000699.

[nur70102-bib-0004] Babapour, A.‐R. , N. Gahassab‐Mozaffari , and A. Fathnezhad‐Kazemi . 2022. “Nurses' Job Stress and Its Impact on Quality of Life and Caring Behaviors: A Cross‐Sectional Study.” BMC Nursing 21, no. 1: 75. 10.1186/s12912-022-00852-y. 35361204 PMC8968092

[nur70102-bib-0005] Beck, C. T. 2011. “Secondary Traumatic Stress in Nurses: A Systematic Review.” Archives of Psychiatric Nursing 25, no. 1: 1–10. 10.1016/j.apnu.2010.05.005. 21251596

[nur70102-bib-0006] Braun, B. , C. Weinland , J. Kornhuber , and B. Lenz . 2018. “Religiosity, Guilt, Altruism and Forgiveness in Alcohol Dependence: Results of a Cross‐Sectional and Prospective Cohort Study.” Alcohol and Alcoholism 53, no. 4: 426–434. 29912267 10.1093/alcalc/agy026

[nur70102-bib-0007] Bub, K. , and M. J. J. Lommen . 2017. “The Role of Guilt in Posttraumatic Stress Disorder.” European Journal of Psychotraumatology 8, no. 1: 1407202. 29230272 10.1080/20008198.2017.1407202 PMC5717716

[nur70102-bib-0008] Cabilan, C. J. , and K. Kynoch . 2017. “Experiences of and Support for Nurses as Second Victims of Adverse Nursing Errors: A Qualitative Systematic Review.” JBI Database of Systematic Reviews and Implementation Reports 15, no. 9: 2333–2364. 10.11124/jbisrir-2016-003254. 28902699

[nur70102-bib-0009] Cohen, S. , T. Kamarck , and R. Mermelstein . 1983. “A Global Measure of Perceived Stress.” Journal of Health and Social Behavior 24: 385–396. 6668417

[nur70102-bib-0010] Conversano, C. , R. Ciacchini , G. Orrù , M. Di Giuseppe , A. Gemignani , and A. Poli . 2020. “Mindfulness, Compassion, and Self‐Compassion Among Health Care Professionals: What's New? A Systematic Review.” Frontiers in Psychology 11: 1683. 10.3389/fpsyg.2020.01683. 32849021 PMC7412718

[nur70102-bib-0011] Dall'Ora, C. , J. Ball , M. Reinius , and P. Griffiths . 2020. “Burnout in Nursing: A Theoretical Review.” Human Resources for Health 18, no. 1: 41. 10.1186/s12960-020-00469-9. 32503559 PMC7273381

[nur70102-bib-0012] Dall'Ora, C. , P. Griffiths , J. Ball , M. Simon , and L. H. Aiken . 2015. “Association of 12 h Shifts and Nurses' Job Satisfaction, Burnout and Intention to Leave: Findings From a Cross‐Sectional Study of 12 European Countries.” BMJ Open 5, no. 9: e008331. https://bmjopen.bmj.com/content/bmjopen/5/9/e008331.full.pdf. 10.1136/bmjopen-2015-008331 PMC4577950 26359284

[nur70102-bib-0013] Dev, V. , A. T. Fernando , and N. S. Consedine . 2020. “Self‐Compassion as a Stress Moderator: A Cross‐Sectional Study of 1700 Doctors, Nurses, and Medical Students.” Mindfulness 11, no. 5: 1170–1181. 10.1007/s12671-020-01325-6. 32435318 PMC7223415

[nur70102-bib-0014] Dolatshad, F. , A. Maher , S. M. Hosseini , and A. Aghili . 2020. “The Correlation of Occupational Stress With Guilt in the Nurses of Mofid Children's Hospital in Tehran, Iran.” Iran Journal of Nursing 33, no. 124: 82–91. 10.29252/ijn.33.124.82.

[nur70102-bib-0015] Duarte, J. , and J. Pinto‐Gouveia . 2017. “Empathy and Feelings of Guilt Experienced by Nurses: A Cross‐Sectional Study of Their Role in Burnout and Compassion Fatigue Symptoms.” Applied Nursing Research 35: 42–47. 10.1016/j.apnr.2017.02.006. 28532725

[nur70102-bib-0016] Duarte, J. , J. Pinto‐Gouveia , and B. Cruz . 2016. “Relationships Between Nurses' Empathy, Self‐Compassion and Dimensions of Professional Quality of Life: A Cross‐Sectional Study.” International Journal of Nursing Studies 60: 1–11. 10.1016/j.ijnurstu.2016.02.015. 27297364

[nur70102-bib-0017] Dyrbye, L. N. , T. D. Shanafelt , P. O. Johnson , L. A. Johnson , D. Satele , and C. P. West . 2019. “A Cross‐Sectional Study Exploring the Relationship Between Burnout, Absenteeism, and Job Performance Among American Nurses.” BMC Nursing 18: 57. 10.1186/s12912-019-0382-7. 31768129 PMC6873742

[nur70102-bib-0018] Fassmer, A. M. , A. Grenz , M. Ennen , et al. 2025. “Perspectives of Healthcare Professionals on Medical Care in Nursing Homes in Germany and The Netherlands: An Explorative Study Using Qualitative Content Analysis.” European Journal of Public Health 35, no. 6: 1191–1197. 10.1093/eurpub/ckaf176. 41016723 PMC12707481

[nur70102-bib-0019] Figley, C. R. 1995. “Compassion Fatigue: Toward a New Understanding of the Costs of Caring.” In Secondary Traumatic Stress: Self Care Issues for Clinicians, Researchers, and Educators, edited by B. H. Stamm . Sidran Press.

[nur70102-bib-0020] Figueiredo‐Ferraz, H. , P. R. Gil‐Monte , E. Grau‐Alberola , and B. Ribeiro do Couto . 2021. “The Mediator Role of Feelings of Guilt in the Process of Burnout and Psychosomatic Disorders: A Cross‐Cultural Study.” Frontiers in Psychology 12: 751211. 10.3389/fpsyg.2021.751211. 35027899 PMC8748256

[nur70102-bib-0021] Goodwin, L. D. , and N. L. Leech . 2006. “Understanding Correlation: Factors That Affect the Size of r.” The Journal of Experimental Education 74, no. 3: 249–266. 10.3200/JEXE.74.3.249-266.

[nur70102-bib-0022] Halpin, S. N. 2024. “Inter‐Coder Agreement in Qualitative Coding: Considerations for Its Use.” American Journal of Qualitative Research 8, no. 3: 23–43. 10.29333/ajqr/14887.

[nur70102-bib-0023] Hashem, Z. , and P. Zeinoun . 2020. “Self‐Compassion Explains Less Burnout Among Healthcare Professionals.” Mindfulness 11: 2542–2551. https://www.ncbi.nlm.nih.gov/pmc/articles/PMC7481342/pdf/12671_2020_Article_1469.pdf. 32929384 10.1007/s12671-020-01469-5 PMC7481342

[nur70102-bib-0024] Hayes, A. F. 2022. *Introduction to Mediation, Moderation, and Conditional Process Analysis: A Regression‐Based Approach* (3rd ed.). The Guilford Press.

[nur70102-bib-0025] Hupfeld, J. , and N. Ruffieux . 2011. “Validierung einer deutschen Version der Self‐Compassion Scale (SCS‐D).” Zeitschrift für klinische Psychologie und Psychotherapie 40, no. 2: 115–123. 10.1026/1616-3443/a000088.

[nur70102-bib-0026] IBM‐Corp . 2023. IBM SPSS Statistics for Windows, Version 29.0 [Computer software]. IBM Corp.

[nur70102-bib-0027] Joinson, C. 1992. “Coping With Compassion Fatigue.” Nursing 22, no. 4: 116–121. 1570090

[nur70102-bib-0028] Jun, J. , M. M. Ojemeni , R. Kalamani , J. Tong , and M. L. Crecelius . 2021. “Relationship Between Nurse Burnout, Patient and Organizational Outcomes: Systematic Review.” International Journal of Nursing Studies 119: 103933. 10.1016/j.ijnurstu.2021.103933. 33901940

[nur70102-bib-0029] Kappes, M. , M. Romero‐García , and P. Delgado‐Hito . 2021. “Coping Strategies in Health Care Providers as Second Victims: A Systematic Review.” International Nursing Review 68, no. 4: 471–481. https://diposit.ub.edu/dspace/bitstream/2445/178985/1/713087.pdf. 34118061 10.1111/inr.12694

[nur70102-bib-0030] Kelly, L. A. , P. M. Gee , and R. J. Butler . 2021. “Impact of Nurse Burnout on Organizational and Position Turnover.” Nursing Outlook 69, no. 1: 96–102. 10.1016/j.outlook.2020.06.008. 33023759 PMC7532952

[nur70102-bib-0031] Klein, E. M. , E. Brähler , M. Dreier , et al. 2016. “The German Version of the Perceived Stress Scale–Psychometric Characteristics in a Representative German Community Sample.” BMC Psychiatry 16, no. 159: 159. 10.1186/s12888-016-0875-9. 27216151 PMC4877813

[nur70102-bib-0032] Lewis, C. L. , J. Langhinrichsen‐Rohling , C. N. Selwyn , and E. C. Lathan . 2019. “Once BITTEN, Twice Shy: An Applied Trauma‐Informed Healthcare Model.” Nursing Science Quarterly 32, no. 4: 291–298. 10.1177/0894318419864344. 31514618

[nur70102-bib-0033] Lim, D. , and D. DeSteno . 2023. “Guilt Underlies Compassion Among Those Who Have Suffered Adversity.” Emotion (Washington, D.C.) 23, no. 3: 613–621. 10.1037/emo0001116. 35951385

[nur70102-bib-0034] LimeSurvey . (n.d.). LimeSurvey: An Open Source Survey Tool. LimeSurvey GmbH.

[nur70102-bib-0035] Losada, A. , M. Márquez‐González , C. Peñacoba , and R. Romero‐Moreno . 2010. “Development and Validation of the Caregiver Guilt Questionnaire.” International Psychogeriatrics 22, no. 4: 650–660. 20170587 10.1017/S1041610210000074

[nur70102-bib-0036] Luo, Y. , R. Meng , J. Li , B. Liu , X. Cao , and W. Ge . 2019. “Self‐Compassion May Reduce Anxiety and Depression in Nursing Students: A Pathway Through Perceived Stress.” Public Health 174: 1–10. 10.1016/j.puhe.2019.05.015. 31265974

[nur70102-bib-0037] McHugh, M. L. 2012. “Interrater Reliability: The Kappa Statistic.” Biochemia Medica 22, no. 3: 276–282. 23092060 PMC3900052

[nur70102-bib-0038] Mira, J. J. , I. Carrillo , S. Lorenzo , et al. 2015. “The Aftermath of Adverse Events in Spanish Primary Care and Hospital Health Professionals.” BMC Health Services Research 15: 151. 10.1186/s12913-015-0790-7. 25886369 PMC4394595

[nur70102-bib-0039] Mottaghi, S. , H. Poursheikhali , and L. Shameli . 2019. “Empathy, Compassion Fatigue, Guilt and Secondary Traumatic Stress in Nurses.” Nursing Ethics 27, no. 2: 494–504. 10.1177/0969733019851548. 31284826

[nur70102-bib-0040] Mróz, J. , and W. Sornat . 2023. “Shame‐ and Guilt‐Proneness and Self‐Compassion as Predictors of Self‐Forgiveness.” Journal of Beliefs & Values 44, no. 2: 188–202. 10.1080/13617672.2022.2076455.

[nur70102-bib-0041] Muchinsky, P. M. 1996. “The Correction for Attenuation.” Educational and Psychological Measurement 56, no. 1: 63–75. 10.1177/0013164496056001004.

[nur70102-bib-0042] Neff, K. D. 2003. “The Development and Validation of a Scale to Measure Self‐Compassion.” Self and Identity 2, no. 3: 223–250. 10.1080/15298860309027.

[nur70102-bib-0043] Neff, K. D. 2023. “Self‐Compassion: Theory, Method, Research, and Intervention.” Annual Review of Psychology 74: 193–218. 10.1146/annurev-psych-032420-031047. 35961039

[nur70102-bib-0044] Norful, A. A. , K. C. Brewer , K. M. Cahir , and A. M. Dierkes . 2024. “Individual and Organizational Factors Influencing Well‐Being and Burnout Amongst Healthcare Assistants: A Systematic Review.” International Journal of Nursing Studies Advances 6: 100187. 10.1016/j.ijnsa.2024.100187. 38746791 PMC11080559

[nur70102-bib-0045] O'Connor, L. E. , J. W. Berry , J. Weiss , M. Bush , and H. Sampson . 1997. “Interpersonal Guilt: The Development of a New Measure.” Journal of Clinical Psychology 53, no. 1: 73–89. 10.1002/(SICI)1097-4679(199701)53:1<73::AID-JCLP10>3.0.CO;2-I. 9120036

[nur70102-bib-0046] Ondrejková, N. , and J. Halamová . 2022. “Prevalence of Compassion Fatigue Among Helping Professions and Relationship to Compassion for Others, Self‐Compassion and Self‐Criticism.” Health & Social Care in the Community 30, no. 5: 1680–1694. 10.1111/hsc.13741. 35133041

[nur70102-bib-0047] Ozeke, O. , V. Ozeke , O. Coskun , and I. I. Budakoglu . 2019. “Second Victims in Health Care: Current Perspectives.” Advances in Medical Education and Practice 10, no. null: 593–603. 10.2147/AMEP.S185912. 31496861 PMC6697646

[nur70102-bib-0048] Panagioti, M. , K. Khan , R. N. Keers , et al. 2019. “Prevalence, Severity, and Nature of Preventable Patient Harm Across Medical Care Settings: Systematic Review and Meta‐Analysis.” BMJ 366: l4185. 10.1136/bmj.l4185. 31315828 PMC6939648

[nur70102-bib-0049] Petitmengin, C. 2006. “Describing One's Subjective Experience in the Second Person: An Interview Method for the Science of Consciousness.” Phenomenology and the Cognitive Sciences 5, no. 3: 229–269. 10.1007/s11097-006-9022-2.

[nur70102-bib-0050] Praxmarer‐Fernande, S. , C. B. Maier , A. Oikarainen , J. Buchan , G. Perfilieva , and W. H. Organization . 2017. “Levels of Education Offered in Nursing and Midwifery Education in the WHO European Region: Multicountry Baseline Assessment.” Public Health Panorama 3, no. 03: 419–430.

[nur70102-bib-0051] Rädiker, S. , and U. Kuckartz . 2019. Analyse Qualitativer Daten Mit MAXQDA. Springer.

[nur70102-bib-0052] Raes, F. , E. Pommier , K. D. Neff , and D. Van Gucht . 2011. “Construction and Factorial Validation of a Short Form of the Self‐Compassion Scale.” Clinical Psychology & Psychotherapy 18, no. 3: 250–255. 10.1002/cpp.702. 21584907

[nur70102-bib-0053] Ruiz‐Fernández, M. D. , R. Ortíz‐Amo , Á. M. Ortega‐Galán , O. Ibáñez‐Masero , M. M. Rodríguez‐Salvador , and J. D. Ramos‐Pichardo . 2020. “Mindfulness Therapies on Health Professionals.” International Journal of Mental Health Nursing 29, no. 2: 127–140. 10.1111/inm.12652. 31498549

[nur70102-bib-0054] Samaie, G. , and H. A. Farahani . 2011. “Self‐Compassion as a Moderator of the Relationship Between Rumination, Self‐Reflection and Stress.” Procedia‐Social and Behavioral Sciences 30: 978–982. 10.1016/j.sbspro.2011.10.190.

[nur70102-bib-0055] Scaffidi Abbate, C. , R. Misuraca , M. Roccella , L. Parisi , L. Vetri , and S. Miceli . 2022. “The Role of Guilt and Empathy on Prosocial Behavior.” Behavioral Sciences 12, no. 3: 64. https://www.mdpi.com/2076-328X/12/3/64. 35323383 10.3390/bs12030064 PMC8945273

[nur70102-bib-0056] Scheel, A. M. 2022. “Why Most Psychological Research Findings Are Not Even Wrong.” Infant and Child Development 31, no. 1: e2295. 10.1002/icd.2295.

[nur70102-bib-0057] Selwyn, C. N. , and E. Lathan . 2021. “Helping Primary Care Patients Heal Holistically via Trauma‐Informed Care.” Journal for Nurse Practitioners 17, no. 1: 84–86. 10.1016/j.nurpra.2020.06.012.

[nur70102-bib-0058] Seys, D. , A. W. Wu , E. Van Gerven , et al. 2013. “Health Care Professionals as Second Victims After Adverse Events:A Systematic Review.” Evaluation & the Health Professions 36, no. 2: 135–162. 10.1177/0163278712458918. 22976126

[nur70102-bib-0059] Singhal, K. , and S. Chukkali . 2023. “The Role of Guilt‐Shame Proneness and Locus of Control in Predicting Moral Injury Among Healthcare Professionals.” Cogent Psychology 10, no. 1: 2264669. 10.1080/23311908.2023.2264669.

[nur70102-bib-0060] Tangney, J. P. , J. Stuewig , and D. J. Mashek . 2007. “Moral Emotions and Moral Behavior.” Annual Review of Psychology 58: 345–372. 10.1146/annurev.psych.56.091103.070145. PMC3083636 16953797

[nur70102-bib-0061] Trull, T. J. , and U. Ebner‐Priemer . 2014. “The Role of Ambulatory Assessment in Psychological Science.” Current Directions in Psychological Science 23, no. 6: 466–470. 10.1177/0963721414550706. 25530686 PMC4269226

[nur70102-bib-0062] Valdez, A. M. 2024. “Burnout or Exploitation? Resiliency Is Not the Solution.” Journal of Emergency Nursing 50, no. 2: 165–166. 10.1016/j.jen.2023.12.014. 38453336

[nur70102-bib-0063] Valdez, C. E. , and M. M. Lilly . 2019. “Modes of Processing Trauma: Self‐Compassion Buffers Affective Guilt.” Mindfulness 10, no. 5: 824–832. 10.1007/s12671-018-1035-8.

[nur70102-bib-0064] Wasson, R. S. , C. Barratt , and W. H. O'Brien . 2020. “Effects of Mindfulness‐Based Interventions on Self‐Compassion in Health Care Professionals: A Meta‐Analysis.” Mindfulness 11, no. 8: 1914–1934. 10.1007/s12671-020-01342-5. 32421083 PMC7223423

[nur70102-bib-0065] Wu, A. W. 2000. “Medical Error: The Second Victim.” BMJ 320, no. 7237: 726–727. 10.1136/bmj.320.7237.726. 10720336 PMC1117748

[nur70102-bib-0066] Xue, M. , Y. Yuan , H. Chen , et al. 2022. “Perceived Stress and Symptoms of Post‐Traumatic Stress Disorder in Nurses: A Moderated Mediation Model of Maladaptive Cognitive Emotional Regulation and Psychological Capital.” Frontiers in Psychiatry 13: 902558. https://www.ncbi.nlm.nih.gov/pmc/articles/PMC9727242/pdf/fpsyt-13-902558.pdf. 36506450 10.3389/fpsyt.2022.902558 PMC9727242

