# Supplementary material for: Feeling of Guilt, Stress, and Self‐Compassion Among Nursing Staff in the Workplace
Source: Res Nurs Health. 2026 Jul 13;49(5):712–23. doi: 10.1002/nur.70102 (PMC13539685; doi:10.1002/nur.70102)
Supplement: Supplementary file 1 [file NUR-49-712-s001.docx]

**Self-compassion as a buffer: Exploring the impact on the experience**

**of guilt and stress in the nursing profession**

**Table of Contents**

[**Supplement S1 – Additional Figures**	1](#_Toc223897413)

[**Figure 2. Post-hoc Mediation Analysis**	1](#_Toc223897414)

[**Figure S1. Scatter plot for visual inspection after LOESS smoothing**	2](#_Toc223897415)

[**Supplement S2 – Coding scheme for open questions**	2](#_Toc223897416)

[**S2.1 Coding scheme for types of guilt-triggering situations in the workplace**	3](#_Toc223897417)

[**S2.2 Coding scheme for strategies for coping with guilt at work**	6](#_Toc223897418)

[**S2.3 Coding scheme for new ways to deal with guilt in the workplace**	10](#_Toc223897419)

[**Supplement S3 – Interrater Reliability**	13](#_Toc223897420)

[**S3.1 Interrater reliability for types of guilt-triggering situations in the workplace**	13](#_Toc223897421)

[**S3.2 Interrater reliability for strategies for coping with guilt at work**	13](#_Toc223897422)

[**S3.3 Interrater reliability for new ways to deal with guilt in the workplace**	14](#_Toc223897423)

# **Supplement S1 – Additional Figures**

## **Figure 2. Post-hoc Mediation Analysis**

b = -5.49^***^

b = -0.19^***^

Interpersonal Guilt

Perceived Stress

Self-Compassion

n.s.

*Note. *** = p < 0.001*

## **Figure S1. Scatter plot for visual inspection after LOESS smoothing**

# **Supplement S2 – Coding scheme for open questions**

**General instruction for the raters**

Through an online survey, healthcare workers were asked about their subjective experiences and quantitative data was collected using questionnaires on self-compassion, stress and feelings of guilt. Qualitative data was also collected through open-ended questions.

For this rating, the qualitative data from the following questions must be categorised:

- “*Please describe a typical situation of your work life, which induces experiences of guilt in your.*” (see coding scheme S2.1)
- *“How do you cope with feelings of guilt?”* (see coding scheme S2.2)
- “*What do you think is needed to better cope with feelings of guilt in your work context*?” (see coding scheme S2.3)

In the files you will find open responses from all participants and the responses should be categorised accordingly. There are different categories that fall into different overarching topic clusters and have been colour coded accordingly. Each category has its own column in the Excel spreadsheet.

**Rules for coding:**

- Please enter a 1 if you think the answer fits the category.
- Please enter a 0 if you think the answer does not fit the category.
- If there are several categories that apply to the answer, you can also assign a 1 to several categories per answer.
- If, in your opinion, none of the given categories apply, please use the "Other" column.
- If you have any comments, you can enter them in the last column.

## **S2.1 Coding scheme for types of guilt-triggering situations in the workplace**

**Code 01 – personnel shortage**

- Description:
  - This category describes triggering factors associated with a lack of personnel.
- Example:
  - ID 14: " *Too many seriously ill patients per nurse to care for them properly, especially in the emergency department.*"

**Code 02 – time shortage**

- Description:
  - This category describes triggering factors that are related to a lack of time.
- Example:
  - ID 20: "*Not enough time for necessary care.*"

**Code 03 – unaccomplished tasks**

- Description:
  - This category describes that subjects have unfinished tasks and that these trigger feelings of guilt.
- Example:
  - ID 41: "*As far as not completely achievable.*"

**Code 04 – errors**

- Description:
  - This category should include mistakes that have led to feelings of guilt.
- Example:
  - ID 243: "*Only if I have actually messed something up.*"

**Code 05 – lack of assistance**

- Description:
  - This category should include situations that represent a lack of assistance to patients and lead to feelings of guilt. There may also be overlaps here with the lack of time category. For an answer to be coded for this category, it must be clear that the patients experience a lack of support due to the lack of time.
- Example:
  - ID 243: " *If I have to keep a resident waiting again because I have to get other residents ready first because they have already rung the bell too.*"

**Code 06 – unexpected situations**

- Description:
  - Unexpected situations (e.g. situations that occur suddenly) that lead to feelings of guilt should be mentioned here.
- Example:
  - ID 189: “*If a resident falls while I am on duty, it could have been prevented if I had been standing by?*"

**Code 07 – dying process**

- Description:
  - Situations should be mentioned here that are in some way related to the dying process of patients and lead to the subjects experiencing feelings of guilt.
- Example:
  - ID 188: " *When I have to reanimate patients even though they didn't really want it.*"

**Code 08 – taking time for yourself**

- Description:
  - The aim here is to describe how time for oneself (e.g. holidays) makes the test subjects feel guilty.
- Example:
  - ID 83: "*Holiday, time off, overtime-free.*"

**Code 09 – no feelings of guilt**

- Description:
  - Answers that show that the test subjects do not feel guilty should be taken into account here.
- Example:
  - ID 168: "*I never have feelings of guilt at work.*"

**Code 10 – other**

- Description:
  - This category should ONLY be selected if the answer does not fit into any of the given categories from your point of view.

**Code 11 – no valid entry**

- Description:
  - This category should be selected if no comprehensible words, only numbers or other symbols are given.

## **S2.2 Coding scheme for strategies for coping with guilt at work**

The following categories describe proactive strategies to help deal with feelings of guilt in the work context. In particular, these strategies suggest that the participants themselves become active in order to deal constructively with feelings of guilt.

**Code 01 - reflection**

- Description:
  - Answers that are categorized in this section should indicate that individuals are dealing with feelings of guilt on a mental level, reflecting on the situations, or considering different perspectives.
- Example:
  - ID 12: "*I try to reflect on what happened and possibly find a solution to handle the situation differently next time, or to plan differently and possibly involve others who can provide assistance.*"

**Code 02 – apologies**

- Description:
  - If the test subjects describe that they consciously apologise to the patients or people affected by the situation, the answers should be classified in this category.
- Example:
  - ID 146: "*I apologise to the patients for the situation and explain it to them.*"

**Code 03 – communication**

- Description:
  - Participants look for ways to communicate in order to cope with feelings of guilt. They may communicate with colleagues or other people.
- Example:
  - ID 14: "*Talking about it with others.*"

**Code 04 – self-compassion / acceptance**

- Description:
  - This category describes that participants reduce feelings of guilt by applying acceptance or self-compassion.
- Example:
  - ID 217: "*I try to tell myself that I do what I can.*"

**Code 05 – separate private & professional life**

- Description:
  - This category describes that participants separate private and professional levels in order to deal with feelings of guilt.
- Example:
  - ID 79: "*Drive home and switch off.*"

**Code 06 – distraction / compensation**

- Description:
  - This category describes how participants distract themselves in order to avoid feeling guilty.
- Example:
  - ID 272: "*Distract myself.*"

**Code 07 – suppress**

- Description:
  - This category describes how subjects suppress feelings of guilt and the situations that trigger them.
- Example:
  - ID 57: "*I try to suppress my feelings, I want to be for myself*."

**Code 08 – negative emotions**

- Description:
  - This category is intended to describe negative emotions and emotional states (e.g., anger, helplessness, depression, etc.) that subjects also experience when they perceive guilt.
- Example:
  - ID 142: "*I get angry.*"

**Code 09 – think / ruminate**

- Description:
  - In this category, subjects were asked to describe how they were constantly thinking or ruminating about the situation in order to deal with feelings of guilt.
- Example:
  - ID 108: "*Think a lot, even at home.*"

**Code 10 – harmful behaviour**

- Description:
  - This category describes harmful behaviours (e.g. drinking or bingeing, etc.) that participants use to cope with feelings of guilt.
- Example:
  - ID 259: "*I drink too much alcohol, like most of my colleagues.*"

**Code 11 – no feelings of guilt**

- Description:
  - Answers that show that the test subjects have no feelings of guilt should be taken into account here.
- Example:
  - ID 111: "*I have no feelings of guilt.*"

**Code 12 – other**

- Description:
  - This category should ONLY be selected if the answer does not fit into any of the given categories from your point of view.

**Code 13 – no valid entry**

- Description:
  - This category should be selected if no comprehensible words, only numbers or other symbols are given.

## **S2.3 Coding scheme for new ways to deal with guilt in the workplace**

**Code 01 – system changes**

- Description:
  - Responses in this category indicate that improvements in system structures are needed.
- Example:
  - ID 11: "*Better healthcare system so that you don't feel bad about being ill.*"

**Code 02 – offers for dialogue**

- Description:
  - Responses in this category should indicate that the suggestions relate to opportunities for dialogue with staff.
- Example:
  - ID 63: "*Open conversations and discussions about how processes can be organised differently. Courage to change and experiment.*"

**Code 03 – support, appreciation & understanding**

- Description:
  - This category includes answers that demand more appreciation and understanding from the system (e.g. employers) for nursing staff.
- Example:
  - ID 118: "*More acceptance of management positions. No "coercion" by talking to people's conscience.*"

**Code 04 – further education**

- Description:
  - Responses in this category should indicate that proposals relate to staff training programmes.
- Example:
  - ID 99: "*More positive experiences. Coaching, motivational training."*

**Code 05 – opportunity for reflection**

- Description:
  - Answers in this category call for more opportunities for reflection in the work context. Reflection can take place within the team or there can also be spaces for individual reflection.
- Example:
  - ID 151: "*Reflection in the team.*"

**Code 06 – find balance**

- Description:
  - Suggestions in this category call for more equalisation at various levels.
- Example:
  - ID 73: "*Work-life balance, enough compensation.*"

**Code 07 – acceptance of the situation and mistakes / self-compassion**

- Description:
  - Responses in this category suggest that more acceptance and self-compassion could help to cope with feelings of guilt.
- Example:
  - ID 16: "*That mistakes can and must happen.*"

**Code 08 – no feelings of guilt / no suggestions**

- Description:
  - Answers assigned to this category should indicate that no feelings of guilt are felt by the respondents.
- Example:
  - ID 22: "*I have no feelings of guilt about my work.*"

**Code 9 – other**

- Description:
  - This category should ONLY be selected if the answer does not fit into any of the given categories from your point of view.

**Code 10 – no valid entry**

- Description:
  - This category should be selected if no comprehensible words, only numbers or other symbols are given.

# **Supplement S3 – Interrater Reliability**

## **S3.1 Interrater reliability for types of guilt-triggering situations in the workplace**

| **Code** | **Cohen’s Kappa** | **95% CI**  **lower bound** | **95% CI upper bound** |
| --- | --- | --- | --- |
| C01 – personnel shortage | 0,92 | 0,84 | 1,01 |
| C02 – time shortage | 0,86 | 0,76 | 0,95 |
| C03 – unaccomplished tasks | 0,60 | 0,41 | 0,79 |
| C04 – errors | 0,82 | 0,65 | 0,99 |
| C05 – lack of assistance | 0,69 | 0,55 | 0,83 |
| C06 – unexpected situations | 0,59 | 0,22 | 0,95 |
| C07 – dying process | 1,0 | 1,0 | 1,0 |
| C08 – taking time for yourself | 0,72 | 0,48 | 0,95 |
| C09 – no feelings of guilt | 0,95 | 0,85 | 1,05 |
| C10 – other | 0,64 | 0,45 | 0,83 |
| C11 – no valid entry | 0,92 | 0,76 | 1,08 |

## **S3.2 Interrater reliability for strategies for coping with guilt at work**

| **Code** | **Cohen’s Kappa** | **95% CI**  **lower bound** | **95% CI upper bound** |
| --- | --- | --- | --- |
| C01 – reflection | 0,35 | 0,06 | 0,65 |
| C02 – apologies | 0,85 | 0,64 | 1,05 |
| C03 – communication | 0,96 | 0,92 | 1,01 |
| C04 – self-compassion / acceptance | 0,90 | 0,77 | 1,04 |
| C05 – separate private & professional life | 0,66 | 0,22 | 1,10 |
| C06 – distraction / compensation | 0,85 | 0,67 | 1,02 |
| C07 – suppress | 0,96 | 0,88 | 1,04 |
| C08 – negative emotions | 0,66 | 0,44 | 0,88 |
| C09 – think / ruminate | 0,83 | 0,64 | 1,02 |
| C10 – harmful behaviour | 0,20 | -0,04 | 0,44 |
| C11 – no feelings of guilt | 0,92 | 0,76 | 1,08 |
| C12 – other | 0,56 | 0,36 | 0,76 |
| C13 – no valid entry | 1,0 | 1,0 | 1,0 |

## **S3.3 Interrater reliability for new ways to deal with guilt in the workplace**

| **Code** | **Cohen’s Kappa** | **95% CI**  **lower bound** | **95% CI upper bound** |
| --- | --- | --- | --- |
| C01 – system changes | 0,88 | 0,80 | 0,97 |
| C02 – offers for dialogue | 0,91 | 0,82 | 1,00 |
| C03 – support, appreciation & understanding | 0,74 | 0,57 | 0,91 |
| C04 – further education | 0,76 | 0,50 | 1,02 |
| C05 – opportunity for reflection | 0,81 | 0,60 | 1,02 |
| C06 – find balance | 0,80 | 0,41 | 1,19 |
| C07 – acceptance of the situation and mistakes / self-compassion | 0,53 | 0,24 | 0,81 |
| C08 – no feelings of guilt / no suggestions | 0,27 | 0,03 | 0,52 |
| C09 – other | 0,30 | 0,12 | 0,47 |
| C10 – no valid entry | 1,0 | 1,0 | 1,0 |
